# Association Between Preoperative Benzodiazepine Use and Postoperative Opioid Use and Health Care Costs

**DOI:** 10.1001/jamanetworkopen.2020.18761

**Published:** 2020-10-27

**Authors:** Chris A. Rishel, Yuting Zhang, Eric C. Sun

**Affiliations:** 1 Department of Anesthesiology, Perioperative and Pain Medicine, Stanford University School of Medicine, Stanford, California; 2 Melbourne Institute: Applied Economic & Social Research, Faculty of Business & Economics, University of Melbourne, Melbourne, Victoria, Australia; 3 Department of Health Research and Policy, Stanford University School of Medicine, Stanford, California

## Abstract

**Importance:**

The association between preoperative benzodiazepine use and long-term postoperative outcomes is not well understood.

**Objective:**

To characterize the association between preoperative benzodiazepine use and postoperative opioid use and health care costs.

**Design, Setting, and Participants:**

In this cohort study, retrospective analysis of private health insurance claims data on 946 561 opioid-naive patients (no opioid prescriptions filled in the year before surgery) throughout the US was conducted. Patients underwent 1 of 11 common surgical procedures between January 1, 2004, and December 31, 2016; data analysis was performed January 9, 2020.

**Exposures:**

Benzodiazepine use, defined as long term (≥10 prescriptions filled or ≥120 days supplied in the year before surgery) or intermittent (any use not meeting the criteria for long term).

**Main Outcomes and Measures:**

The primary outcome was opioid use 91 to 365 days after surgery. Secondary outcomes included opioid use 0 to 90 days after surgery and health care costs 0 to 30 days after surgery.

**Results:**

In this sample of 946 561 patients, the mean age was 59.8 years (range, 18-89 years); 615 065 were women (65.0%). Of these, 23 484 patients (2.5%) met the criteria for long-term preoperative benzodiazepine use and 47 669 patients (5.0%) met the criteria for intermittent use. After adjusting for confounders, long-term (odds ratio [OR], 1.59; 95% CI, 1.54-1.65; *P* < .001) and intermittent (OR, 1.47; 95% CI, 1.44-1.51; *P* < .001) benzodiazepine use were associated with an increased probability of any opioid use during postoperative days 91 to 365. For patients who used opioids in postoperative days 91 to 365, long-term benzodiazepine use was associated with a 44% increase in opioid dose (additional 0.6 mean daily morphine milligram equivalents [MMEs]; 95% CI, 0.3-0.8 MMEs; *P* < .001), although intermittent benzodiazepine use was not significantly different (0.0 average daily MMEs; 95% CI, −0.2 to 0.2 MMEs; *P* = .65). Preoperative benzodiazepine use was also associated with increased opioid use in postoperative days 0 to 90 for both long-term (32% increase, additional 1.9 average daily MMEs; 95% CI, 1.6-2.1 MMEs; *P* < .001) and intermittent (9% increase, additional 0.5 average daily MMEs; 95% CI, 0.4-0.6 MMEs; *P* < .001) users. Intermittent benzodiazepine use was associated with an increase in 30-day health care costs ($1155; 95% CI, $938-$1372; *P* < .001), while no significant difference was observed for long-term benzodiazepine use.

**Conclusions and Relevance:**

The findings of this study suggest that, among opioid-naive patients, preoperative benzodiazepine use may be associated with an increased risk of developing long-term opioid use and increased opioid dosages postoperatively, and also may be associated with increased health care costs.

## Key Points

**Question:**

Is preoperative benzodiazepine use associated with increased postoperative opioid use and health care costs for opioid-naive patients undergoing surgery?

**Findings:**

In this US national cohort study of 945 561 opioid-naive patients undergoing 1 of 11 surgical procedures, an increased risk for long-term opioid use postoperatively was noted in patients with both long-term and intermittent preoperative benzodiazepine use compared with benzodiazepine-naive patients. Some associations between preoperative benzodiazepine use and increased postoperative opioid dosages and health care costs were also observed.

**Meaning:**

The findings of this study suggest that, among opioid-naive patients undergoing surgery, preoperative benzodiazepine use may increase the risk of postoperative long-term opioid use and may increase health care costs.

## Teaser

This cohort study analyzes the preoperative use of benzodiazepines compared with long-term postoperative use of opioids and association with health care costs.

## Introduction

Opioid use remains a public health issue in the US. Between 1999 and 2017, over 399 000 people died of opioid-related drug overdoses in the US.^1,2^ Since 1999, the annual rate of overdose death involving prescription opioids has increased by a factor of 5.^1^ Numerous opioid-related studies have focused on the perioperative period, which is a time with inherent risk to develop long-term opioid use postoperatively.^3^ Opioids are frequently used to treat acute postoperative surgical pain, with more than 80% of patients being prescribed opioids after low-risk surgery.^4^ Furthermore, long-term preoperative opioid use has been associated with worse perioperative outcomes, including increased mortality, costs, longer hospital lengths of stay, higher rates of surgical complications, and more frequent readmissions, as well as increased postoperative opioid use.^5,6,7^

Benzodiazepines are another commonly prescribed class of medications that carry risk of dependence and overdose despite receiving significantly less attention than opioids,^8^ and the consequences of benzodiazepine use in the perioperative period and how benzodiazepine use influences postoperative opioid use are less well understood. In 2008, it was estimated that 5.2% of adults used benzodiazepines, and that use of the drugs increased with age to as high as 8.7% among adults aged 65 to 80 years.^9^

Research has shown that concurrent use of benzodiazepines and opioids is independently associated with an increased risk for an emergency department visit due to overdose and that preoperative use of medications from both classes is associated with increased mortality after noncardiac surgery.^10,11^ In addition, several studies^12,13,14^ have suggested an association between preoperative benzodiazepine use and an increased risk for postoperative adverse events, and one single-center study^11^ from Iceland identified an association with increased postoperative opioid use. However, the association between long-term use and amount of benzodiazepine being used preoperatively and persistent postoperative opioid use, as well as the association with postoperative health care costs, remain not well understood. Therefore, this study used a national database of US health care claims to examine whether preoperative benzodiazepine use was associated with an increased risk for long-term opioid use and increased health care costs among patients who did not use opioids preoperatively. A secondary analysis explored the association of duration and dosage of benzodiazepines with long-term postoperative opioid use.

## Methods

### Data Source and Population

The data consist of deidentified administrative health claims from the US (Clinformatics Data Mart Database, Optum). The database includes approximately 17 million to 19 million annual covered lives for a total of over 57 million unique lives. These administrative claims submitted for payment by providers and pharmacies are verified, adjudicated, adjusted, and deidentified before inclusion. The data for inpatient and outpatient encounters provide diagnosis codes, procedure codes, and dates of service (eTable 1 in the Supplement). Pharmacy claims data provide the generic name of the drug, fill date, quantity supplied, and number of days supplied. This study received institutional review board approval from Stanford University, which also issued a waiver of consent because the data are deidentified. This study followed the Strengthening the Reporting of Observational Studies in Epidemiology (STROBE) reporting guideline.

The initial sample included 3 624 573 surgical procedures between January 1, 2004, and December 31, 2016, for patients aged 18 to 90 years undergoing 1 of the following procedures: total knee arthroplasty, total hip arthroplasty, laparoscopic cholecystectomy, open cholecystectomy, laparoscopic appendectomy, open appendectomy, cesarean delivery, functional endoscopic sinus surgery, cataract surgery, transurethral resection of the prostate, or simple mastectomy. We excluded patients who underwent any other procedure in the year before or after the operation of interest, except for postoperative days 0 to 30 to measure postoperative complications (n = 669 601). We then excluded patients who did not have continuous enrollment in their insurance plan during this 2-year perioperative window (n = 1 647 628). From this population of 1 307 344 patients, we excluded those who were prescribed any opioid in the preoperative year (n = 360 783 [27.6%]), although opioid prescriptions in the 7 days before surgery were not considered since patients may prefill their postoperative prescriptions. The final sample consisted of 946 561 patients (eFigure 1 in the Supplement).

### Outcomes

The primary outcome was whether patients continued to use opioids during postoperative days 91 to 365 and, if so, the mean daily dose prescribed. Secondary outcomes included the average daily dose of opioid prescribed during postoperative days 0 to 90 and total health care costs during the first 30 days after surgery. The time windows for postoperative opioid use were chosen to differentiate between treatment of acute postoperative pain and pain that persists beyond the expected recovery period. Health care costs were calculated as the sum of the amount billed to the patient’s insurer.

For our opioid analyses, we included prescriptions for codeine, dihydrocodeine, fentanyl, hydrocodone, hydromorphone, meperidine, methadone, morphine, oxycodone, and oxymorphone. Each prescription was converted to an average daily dose in oral morphine equivalents (MMEs) using the prescription date, generic name, strength, number of units dispensed, and number of days supplied provided in the database.^15^

### Exposure

The independent variable of interest was whether the patient used benzodiazepines in the year before surgery. Patients using benzodiazepines were classified as either long-term users (defined as having ≥10 prescriptions filled or ≥120 days prescribed in the year before surgery) or intermittent users (defined as >0 and <10 prescriptions filled or >0 and <120 days prescribed). These patients were compared with patients who had no benzodiazepines prescribed in the year before surgery.

For our benzodiazepine analyses, we included prescriptions for alprazolam, chlordiazepoxide, clonazepam, clorazepate, diazepam, estazolam, flurazepam, lorazepam, oxazepam, quazepam, temazepam, and triazolam. While oral MMEs are a well-studied and commonly used tool for clinical practice and research use,^15,16^ evidence describing benzodiazepine equivalence has been limited, with some inconsistencies between studies.^17,18,19,20,21^ However, the US Department of Health and Human Services Center for Substance Abuse Treatment has published a protocol that aggregates these data and provides conversions for the major oral benzodiazepines prescribed in the US.^22^ Using these data, we developed a tool—oral diazepam milligram equivalents (DMEs)—to approximately quantify benzodiazepine use and converted each prescription in the data to an average daily dose of benzodiazepine in DMEs (eTable 2 in the Supplement).

### Variables

Additional variables were included to adjust for potential confounding. Age, sex, and year of surgery were directly extracted from the claims data. We used previously described methods^23^ to identify medical comorbidities included in the Elixhauser index.^24^ While the Elixhauser index includes comorbidities that are related to benzodiazepine use (eg, neurologic disorders, alcohol abuse, drug abuse, psychoses, and depression), anxiety disorders are not included. Thus, we supplied an additional variable to adjust for anxiety disorders.^25^ Comorbidities were identified using diagnosis codes and were included if a relevant code appeared between when the patient was first enrolled in their insurance plan and the date of surgery. Variables were also included for each type of surgery in the analysis to adjust for the procedure being performed.

### Statistical Analysis

We analyzed demographic and comorbidity data using descriptive statistics, including means and 95% CIs. To assess for differences in characteristics between patients who used benzodiazepines preoperatively and those who did not, we performed 2-sample *t* tests for continuous variables (eg, age) and χ^2^ tests for categorical variables (eg, sex, comorbidities, and type of procedure). Findings were considered significant at *P* < .01.

We then estimated the association between preoperative benzodiazepine use and our outcomes using multivariable regression models, including adjustments for potential confounders of age, sex, year of surgery, type of surgery, and patient comorbidities.

For our primary outcome of opioid use in postoperative days 91 to 365, 812 549 of the patients in our sample (85.8%) did not have any opioid prescribed in this period. When there are many observations for which the dependent variable—in this case, opioid use during postoperative days 91 to 365—is zero, a simple linear regression analysis will tend to be downward-biased (ie, the estimated effect will be lower in magnitude than the true effect).^26^ Therefore, we performed a 2-step analysis. The first analysis consisted of multivariable logistic regression in which the dependent variable was whether the patient used any opioid in postoperative days 91 to 365 and the 2 independent variables of interest were whether the patient was classified as having long-term or intermittent benzodiazepine use preoperatively, which estimates the association between preoperative benzodiazepine use and whether the patient subsequently used any opioid.

The second analysis consisted of a multivariable linear regression in which the dependent variable was the amount of opioid prescribed in average daily MMEs during postoperative days 91 to 365, and our independent variable of interest was whether the patient was classified as having long-term or intermittent use of benzodiazepines preoperatively. This analysis was restricted to patients who used at least some opioid during postoperative days 91 to 365 and estimated the association between preoperative benzodiazepine use and the amount of opioid used during that postoperative period. This 2-step approach has been used in other studies to obtain nonbiased estimates when many observations assume a value of 0.^27,28^

For the secondary outcomes, we conducted multivariable linear regression analyses with the same set of covariates as used for the baseline model described above. The analyses were performed on January 9, 2020, using MATLAB, version R2015a (MathWorks Inc).

### Sensitivity Analyses

To measure differences in our primary outcomes for each type of surgery, we performed a subgroup analysis, repeating the main analyses for each operation independently. Furthermore, because our definitions of benzodiazepine use are both arbitrary and dichotomous, we performed a sensitivity analysis to evaluate the robustness of the results using the DME technique described in the Exposure subsection and in eTable 2 in the Supplement. We calculated the average daily dose of benzodiazepines in the preoperative year for each patient in the sample and repeated the regression analyses described above for the primary and secondary outcomes, substituting the dichotomous long-term or intermittent benzodiazepine cohort variable for the average daily DME as a continuous variable.

## Results

The final sample consisted of 946 561 patients; of these, 615 065 patients (65.0%) were women and 331 496 patients (35.0%) were men; mean age was 59.8 years (range, 18-89 years). The patients were undergoing 1 of 11 surgical procedures with no opioid prescriptions in the year before surgery. Of the 946 561 patients, 71 153 patients (7.5%) were prescribed some benzodiazepine in the preoperative year, with 23 484 patients (2.5%) meeting the criteria to be classified as long-term benzodiazepine users and 47 669 patients (5.0%) classified as intermittent benzodiazepine users (eFigure 2 in the Supplement). The remaining 875 408 patients (92.5%) were classified as non–benzodiazepine users. Patients who were prescribed benzodiazepines preoperatively were older, more likely to be women, and had a higher incidence of many comorbidities. There were also differences in the percentage of benzodiazepine users undergoing several procedures compared with non–benzodiazepine users (Table 1).

**Table 1.** Sample Characteristics^a^

Variable	Benzodiazepine-naive patients, % (SE) (n = 875 408)	Patients with preoperative benzodiazepine use			
		Long-term (n = 23 484)		Intermittent (n = 47 669)	
		% (SE)	*P* value^b^	% (SE)	*P* value^b^
Demographic characteristics					
Age, mean (SE), y	59.6 (0.0)	66.1 (0.1)	<.001	59.3 (0.1)	<.001
Women	64.3 (0.0)	70.7 (0.2)	<.001	75.4 (0.2)	<.001
Year of surgery, mean (SE)	2010.9 (0.0)	2011.6 (0.0)	<.001	2011.2 (0.0)	<.001
Narcotic use in preoperative year					
Benzodiazepine prescription, No. (SE)	0	7.9 (0.0)	<.001	1.7 (0.0)	<.001
Days with benzodiazepine prescription, No. (SE)	0	249 (1)	<.001	32.8 (0.1)	<.001
Average daily diazepam milligram equivalents prescribed	0	7.7 (0.1)	<.001	0.8 (0.0)	<.001
Type of surgery					
Total arthroplasty					
Knee	6.4 (0.0)	7.7 (0.2)	<.001	6.2 (0.1)	.04
Hip	3.5 (0.0)	3.4 (0.1)	.52	3.4 (0.1)	.42
Cholecystectomy					
Laparoscopic	12.2 (0.0)	15.5 (0.2)	<.001	17.7 (0.2)	<.001
Open	0.6 (0.0)	0.7 (0.1)	.08	0.6 (0.0)	.31
Appendectomy					
Laparoscopic	4.2 (0.0)	3.0 (0.1)	<.001	3.8 (0.1)	<.001
Open	0.9 (0.0)	0.7 (0.1)	<.001	0.7 (0.0)	<.001
Cesarean delivery	16.7 (0.0)	1.5 (0.1)	<.001	9.9 (0.1)	<.001
Functional endoscopic sinus surgery	4.3 (0.0)	5.3 (0.1)	<.001	7.3 (0.1)	<.001
Cataract surgery	47.2 (0.1)	57.3 (0.3)	<.001	41.2 (0.2)	<.001
Transurethral resection of the prostate	1.3 (0.0)	1.3 (0.1)	.88	1.2 (0.0)	.006
Simple mastectomy	2.5 (0.0)	3.7 (0.1)	<.001	8.0 (0.1)	<.001
Comorbidities					
Congestive heart failure	7.5 (0.0)	11.7 (0.2)	<.001	8.1 (0.1)	<.001
Cardiac arrhythmias	18.7 (0.0)	28.7 (0.3)	<.001	23.1 (0.2)	<.001
Valvular disease	12.6 (0.0)	20.1 (0.3)	<.001	16.1 (0.2)	<.001
Pulmonary circulation disorders	2.8 (0.0)	5.1 (0.1)	<.001	3.3 (0.1)	<.001
Peripheral vascular disorders	12.8 (0.0)	19.6 (0.3)	<.001	13.9 (0.2)	<.001
Hypertension					
Uncomplicated	54.5 (0.1)	72.7 (0.3)	<.001	59.0 (0.2)	<.001
Complicated	9.7 (0.0)	14.5 (0.2)	<.001	10.5 (0.1)	<.001
Paralysis	0.9 (0.0)	1.7 (0.1)	<.001	1.2 (0.0)	<.001
Other neurologic disorders	4.8 (0.0)	11.1 (0.2)	<.001	7.2 (0.1)	<.001
Chronic pulmonary disease	22.4 (0.0)	36.3 (0.3)	<.001	29.1 (0.2)	<.001
Diabetes					
Uncomplicated	22.8 (0.0)	27.3 (0.3)	<.001	21.6 (0.2)	<.001
Complicated	9.6 (0.0)	11.5 (0.2)	<.001	8.7 (0.1)	<.001
Hypothyroidism	20.6 (0.0)	31.4 (0.3)	<.001	27.2 (0.2)	<.001
Kidney failure	8.8 (0.0)	12.0 (0.2)	<.001	8.2 (0.1)	<.001
Liver disease	7.8 (0.0)	13.0 (0.2)	<.001	11.5 (0.1)	<.001
Peptic ulcer disease	1.8 (0.0)	3.5 (0.1)	<.001	2.7 (0.1)	<.001
AIDS/HIV	0.2 (0.0)	0.3 (0.0)	<.001	0.2 (0.0)	<.001
Lymphoma	0.8 (0.0)	1.3 (0.1)	<.001	1.1 (0.0)	<.001
Metastatic cancer	1.3 (0.0)	2.2 (0.1)	<.001	3.0 (0.1)	<.001
Solid tumor without metastases	11.2 (0.0)	15.8 (0.2)	<.001	16.6 (0.2)	<.001
Rheumatoid arthritis/collagen vascular diseases	6.4 (0.0)	11.8 (0.2)	<.001	9.3 (0.1)	<.001
Coagulopathy	3.7 (0.0)	5.1 (0.1)	<.001	4.6 (0.1)	<.001
Obesity	14.3 (0.0)	16.8 (0.2)	<.001	17.0 (0.2)	<.001
Weight loss	4.8 (0.0)	10.2 (0.2)	<.001	7.0 (0.1)	<.001
Fluid and electrolyte disorder	12.9 (0.0)	23.0 (0.3)	<.001	16.9 (0.2)	<.001
Blood loss anemia	1.7 (0.0)	2.9 (0.1)	<.001	2.0 (0.1)	<.001
Deficiency anemia	7.6 (0.0)	13.5 (0.2)	<.001	9.8 (0.1)	<.001
Alcohol abuse	1.7 (0.0)	4.3 (0.1)	<.001	3.1 (0.1)	<.001
Drug abuse	0.9 (0.0)	4.3 (0.1)	<.001	2.1 (0.1)	<.001
Psychosis	1.5 (0.0)	6.2 (0.2)	<.001	3.2 (0.1)	<.001
Depression	14.8 (0.0)	48.4 (0.3)	<.001	35.8 (0.2)	<.001
Anxiety disorder	13.5 (0.0)	59.8 (0.3)	<.001	48.1 (0.2)	<.001

^a^ Overall, patients with preoperative benzodiazepine use were older, more likely to be women, and had a higher incidence of many comorbidities.

^b^ *P* values reflect the comparison between their benzodiazepine cohort and benzodiazepine-naive patients and were computed using χ^2^ for categorical variables and 2-sample *t* tests for continuous variables.

### Main Analysis

Before adjustment for observed confounders, opioids were more likely to be prescribed in postoperative days 91 to 365 for both long-term (22.4%; 95% CI, 21.9%-23.0%; *P* < .001) and intermittent (21.1%; 95% CI, 20.8%-21.5%; *P* < .001) benzodiazepine users compared with non–benzodiazepine users (13.6%; 95% CI, 13.5%-13.6%). In addition, among patients continuing to use opioids in this period, long-term benzodiazepine users were prescribed higher opioid doses on average (3.9 average daily MMEs; 95% CI, 3.6-4.2 MMEs; *P* < .001) than non–benzodiazepine users (2.7 average daily MMEs; 95% CI, 2.6-2.7 MMEs), although no significant difference was found for intermittent benzodiazepine users (2.9 average daily MMEs; 95% CI, 2.7-3.0 MMEs; *P* = .03).

After adjusting for differences in patient characteristics and medical comorbidities, patients who used benzodiazepines in the year before surgery remained significantly more likely to have an opioid prescribed in postoperative days 91 to 365 compared with non–benzodiazepine users (non–benzodiazepine users: adjusted probability, 13.0%; long-term users: adjusted probability, 19.2%; odds ratio [OR], 1.59; 95% CI, 1.54-1.65, *P* < .001; intermittent users: adjusted probability 18.0%; OR, 1.47; 95% CI, 1.44-1.51; *P* < .001). Similarly, for patients continuing to use opioids in this period, long-term benzodiazepine users were prescribed 44% higher opioid dosages, corresponding to an increase of 0.6 (95% CI, 0.3-0.8; *P* < .001) average daily MMEs compared with non–benzodiazepine users. No significant difference was observed between intermittent and non–benzodiazepine users (difference of 0.0 average daily MMEs, 95% CI, −0.2 to 0.2; *P* = .65 MMEs) (Table 2).

**Table 2.** Outcomes^a^

Variable	Benzodiazepine-naive patients	Patients with preoperative benzodiazepine use					
		Long-term			Intermittent		
		Measure (95% CI)	Difference or OR (95% CI)	*P* value	Measure (95% CI)	Difference or OR (95% CI)	*P* value
**Primary outcomes (postoperative days 91-365)**							
Probability of any opioid prescribed, % (95% CI)							
Unadjusted	13.6 (13.5 to 13.6)	22.4 (21.9 to 23.0)	OR, 1.84 (1.79 to 1.90)	<.001	21.1 (20.8 to 21.5)	OR, 1.71 (1.67 to 1.75)	<.001
Adjusted^b^	13.0 (12.9 to 13.1)	19.2 (18.7 to 19.7)	OR, 1.59 (1.54 to 1.65)	<.001	18.0 (17.7 to 18.4)	OR, 1.47 (1.44 to 1.51)	<.001
Average daily opioid prescribed among patients still using opioids, MME (95% CI)^c^							
Unadjusted	2.7 (2.6 to 2.7)	3.9 (3.6 to 4.2)	Difference, 1.2 (0.9 to 1.5)	<.001	2.9 (2.7 to 3.0)	Difference, 0.2 (0.0 to 0.4)	.03
Adjusted^b^	2.7 (2.7 to 2.8)	3.3 (3.0 to 3.6)	Difference, 0.6 (0.3 to 0.8)	<.001	2.7 (2.5 to 2.9)	Difference, 0.0 (−0.2 to 0.2)	.65
**Secondary outcomes**							
Average daily opioid prescribed in postoperative days 0-90, MME (95% CI)^c^							
Unadjusted	5.9 (5.9 to 5.9)	7.8 (7.5 to 8.0)	Difference, 1.9 (1.6 to 2.1)	<.001	6.4 (6.2 to 6.5)	Difference, 0.5 (0.4 to 0.6)	<.001
Adjusted^b^	5.9 (5.9 to 5.9)	6.7 (6.5 to 6.9)	Difference, 0.8 (0.6 to 0.9)	<.001	6.3 (6.2 to 6.4)	Difference, 0.3 (0.2 to 0.4)	<.001
Total health care costs in postoperative days 0-30, $ (95% CI)							
Unadjusted	22 035 (21 977 to 22 093)	22 267 (21 909 to 22 626)	Difference, 232 (−130 to 595)	.20	25 158 (24 876 to 25 440)	Difference, 3123 (2835 to 3411)	<.001
Adjusted^b^	22 138 (22 089 to 22 186)	22 238 (21 938 to 22 538)	Difference, 101 (−204 to 405)	.52	23 293 (23 082 to 23 504)	Difference, 1155 (938 to 1372)	<.001

Abbreviation: MME, morphine milligram equivalents.

^a^ Both long-term and intermittent benzodiazepine use were associated with an increased likelihood to continue to use opioids after surgery and higher opioid dose requirements in the immediate postoperative period. Long-term benzodiazepine use was also associated with increased opioid doses beyond the immediate postoperative period. Intermittent benzodiazepine use was associated with increased 30-day health care costs.

^b^ Results were adjusted for age, sex, type and year of surgery, and medical comorbidities using regression modeling.

^c^ Patients who were not prescribed any opioid in the referenced time period were excluded from the analysis to prevent downward biasing of the results.

For our secondary outcomes, after adjustment, both long-term and intermittent benzodiazepine users were prescribed higher opioid dosages in postoperative days 0 to 90 compared with non–benzodiazepine users (long-term users: 32% increase, difference of 1.9 average daily MMEs; 95% CI, 1.6-2.1 MMEs; *P* < .001; intermittent users: 9% increase, difference of 0.5 average daily MMEs; 95% CI, 0.4-0.6 MMEs; *P* < .001). In addition, a 5% increase in total health care costs during postoperative days 0 to 30 was observed between intermittent and non–benzodiazepine users (difference of $1155; 95% CI, $938-$1372; *P* < .001), although no significant difference was noted for long-term users (difference of $101; 95% CI, −$204 to $405; *P* = .52).

### Sensitivity Analyses

A subgroup analysis stratifying patients by type of surgery reinforced our findings for increased odds of continued opioid use in postoperative days 91 to 365 among patients using benzodiazepines preoperatively (Table 3). However, among patients continuing to use opioids in this period, there were not strong associations between preoperative benzodiazepine use and postoperative opioid doses within each type of surgery.

**Table 3.** Subgroup Analysis of Primary Outcomes by Type of Procedure^a^

Surgical procedure	No. of patients	No. (%) of patients with postoperative long-term opioid use	Patients with preoperative benzodiazepine use						
			Long-term				Intermittent	
			No. (%) of patients	Postoperative long-term opioid use, OR (95% CI)	Difference in average daily dose of opioid during postoperative days 91-365 (95% CI)	No. (%) of patients	Postoperative long-term opioid use, OR (95% CI)	Difference in average daily dose of opioid during postoperative days 91-365 (95% CI)
Total arthroplasty								
Knee	60 867	15 727 (25.8)	1801 (3.0)	1.62 (1.47 to 1.80)^b^	0.7 (−0.3 to 1.7)	2943 (4.8)	1.44 (1.33 to 1.56)^b^	−0.1 (−0.9 to 0.7)
Hip	33 172	5323 (16.0)	806 (2.4)	1.92 (1.62 to 2.27)^b^	1.2 (0.0 to 2.4)	1640 (4.9)	1.48 (1.30 to 1.67)^b^	−0.2 (−1.1 to 0.8)
Cholecystectomy								
Laparoscopic	119 374	19 789 (16.6)	3633 (3.0)	1.62 (1.50 to 1.76)^b^	0.5 (−0.2 to 1.2)	8421 (7.1)	1.48 (1.40 to 1.56)^b^	−0.2 (−0.7 to 0.3)
Open	5793	953 (16.5)	165 (2.8)	1.45 (0.98 to 2.20)	−1.1 (−7.4 to 5.1)	274 (4.7)	1.75 (1.31 to 2.34)^b^	7.8 (3.4 to 12.1)^b^
Appendectomy								
Laparoscopic	39 109	5901 (15.1)	695 (1.8)	1.41 (1.16 to 1.70)^b^	1.6 (0.6 to 2.6)^c^	1829 (4.7)	1.41 (1.25 to 1.59)	0.2 (−0.5 to 0.8)
Open	8508	1228 (14.4)	156 (1.8)	1.31 (0.86 to 1.99)	−1.0 (−5.0 to 3.1)	341 (4.0)	1.58^b^ (1.20 to 2.08)	−0.7 (−3.4 to 1.9)
Cesarean delivery	151 469	18 573 (12.3)	360 (0.2)	2.32 (1.8 to 2.90)^b^	0.7 (−0.4 to 1.9)	4728 (3.1)	1.56 (1.44 to 1.68)^b^	0.9 (0.5 to 1.3)^b^
Functional endoscopic sinus surgery	42 653	8312 (19.5)	1234 (2.9)	1.44 (1.26 to 1.64)^b^	−0.6 (−1.8 to 0.7)	3479 (8.2)	1.37 (1.26 to 1.49)^b^	−0.3 (−1.1 to 0.5)
Cataract surgery	446 613	51 953 (11.6)	13 446 (3.0)	1.59 (1.52 to 1.67)^b^	0.5 (0.1 to 0.9)^c^	19 960 (4.4)	1.49 (1.43 to 1.55)^b^	−0.2 (−0.5 to 0.1)
Transurethral resection of the prostate	12 396	1794 (14.5)	312 (2.5)	NA^d^	1.5 (−0.7 to 3.7)	557 (4.5)	NA^d^	0.2 (−1.5 to 1.8)
Mastectomy	26 607	4456 (16.7)	876 (3.3)	1.73 (1.47 to 2.04)^b^	1.0 (−0.7 to 2.8)	3797 (14.3)	1.36 (1.24 to 1.49)^b^	−0.5 (−1.5 to 0.5)

Abbreviations: NA, not applicable; OR, odds ratio.

^a^ Increased odds of opioid use in postoperative days 91 to 365 for both long-term and intermittent preoperative benzodiazepine users across most surgery types in our sample. However, additional associations between preoperative benzodiazepine use and the dose of postoperative opioid used in the same period was not found.

^b^ *P* < .001.

^c^ *P* < .01.

^d^ Regression models were unable to converge to generate coefficients for the variables of interest.

Substitution of the long-term or intermittent benzodiazepine cohort variable with the average daily dose of benzodiazepine used in the preoperative year in DMEs supported robustness of our primary and secondary outcomes. Significant associations were observed between higher preoperative benzodiazepine doses and increased odds of continuing to use opioids in postoperative days 91 to 365 (OR, 1.36 for a 10-mg increase in average daily DMEs; 95% CI, 1.33-1.39; *P* < .001), as well as increased opioid dosages among patients continuing to use opioids in this period (0.7 average daily MMEs per 10 mg of average daily DMEs; 95% CI, 0.5-0.9 MMEs; *P* < .001).

For our secondary outcomes, there was an association between the amount of benzodiazepines used preoperatively and an increase in the amount of opioids prescribed in postoperative days 0 to 90 among patients using opioids (0.8 average daily MMEs per 10 mg of average daily DMEs; 95% CI, 0.6-0.9 MMEs; *P* < .001). No significant association was observed between the average daily dose of benzodiazepines used preoperatively and the total health care costs in postoperative days 0 to 30 ($10 per 10 mg of average daily DMEs; $99 per 10 mg of average daily DMEs; 95% CI, −$163 to $361; *P* = .46) (Table 4).

**Table 4.** Sensitivity Analysis Results^a^

Variable	Change (95% CI)^b^	*P* value
Primary outcomes (postoperative days 91-365)		
Adjusted OR of any opioid prescribed, %	1.36 (1.33-1.39)	<.001
Average daily opioid prescribed, MME	0.7 (0.5 to 0.9)	<.001
Secondary outcomes (postoperative days 0-90)		
Average daily opioid prescribed, MME	0.8 (0.6 to 0.9)	<.001
Total health care costs, $	99 (−163 to 361)	.46

Abbreviations: DME, diazepam milligram equivalent; MME, morphine milligram equivalent.

^a^ The robustness of the main results of this study was evaluated by measuring the association between the specific dose of benzodiazepine being used preoperatively in DMEs and our outcomes. This sensitivity analysis was mostly consistent with the primary analysis, finding direct associations between preoperative benzodiazepine dose and an increased likelihood of persistent opioid use as well as increased doses of opioids prescribed both in the immediate postoperative period and long term. However, no association between the dose of benzodiazepine and 30-day postoperative health care costs was observed.

^b^ Marginal change in outcome, per 10 mg of average daily DMEs used during preoperative year.

## Discussion

In this retrospective analysis of 946 561 opioid-naive patients aged 18 to 89 years undergoing 1 of 11 surgical procedures, long-term or intermittent benzodiazepine use in the year before surgery was associated with increased odds of continuing to use opioids in postoperative days 91 to 365. Among patients continuing to use opioids in this period, long-term preoperative benzodiazepine use was associated with an increase in the amount of opioid prescribed compared with patients who did not use benzodiazepines preoperatively, although the difference in doses was small and no significant difference was observed for intermittent benzodiazepine use, suggesting limited clinical significance. A secondary analysis that directly quantified preoperative benzodiazepine dosages also showed a positive association between the dose of benzodiazepine used preoperatively and the amount of opioids prescribed in postoperative days 91 to 365.

For our secondary outcomes, preoperative benzodiazepine use was associated with a small increase in the amount of opioids prescribed in postoperative days 0 to 90 among patients who used any opioid in this period compared with benzodiazepine-naive patients. An association between intermittent preoperative benzodiazepine use and a small increase in total health care costs during the first 30 days postoperatively was also observed. However, this association was not found for patients who used benzodiazepines over a long period, nor was this association replicated in our sensitivity analysis.

Our analysis adjusted for multiple potential demographic and medical confounders, including comorbidities related to benzodiazepine use or misuse. The results were robust in a sensitivity analysis that quantified preoperative benzodiazepine use in DMEs rather than including number of prescriptions or prescription-days as a surrogate for benzodiazepine use in the main analysis.

Recent studies have suggested that preoperative benzodiazepine use is associated with worsened surgical outcomes.^11,12^ One study used a nationwide sample from the US with the same primary data source as our study and suggested that preoperative benzodiazepine use is associated with increased postoperative adverse events.^12^ Another single-center study based in Iceland also demonstrated an increased risk for postoperative opioid use and dose-dependent effect on the risk of adverse events associated with preoperative benzodiazepine use.^11^ However, the generalizability of the second study to the US population is unclear given lower rates of comorbidities and lower mean age in their sample, and potential differences in practice habits related to narcotic prescribing between the 2 countries.

To our knowledge, no previous study has assessed how preoperative benzodiazepine use relates to the duration and amount of opioids used, as well as health care costs in the perioperative period. Our study expands on previous work by suggesting an association between the intensity of preoperative benzodiazepine use and an increase in both probability of continuing to use opioids long term and the opioid dose being prescribed. This finding may suggest overlap in the exposures and triggers leading to psychotropic drug dependence. However, the economic implications of preoperative benzodiazepine use may be limited, as the association between preoperative benzodiazepine use and increased postoperative costs was small and inconsistent.

### Limitations

This study has several limitations. First, we found many differences in the sample characteristics between patients who used benzodiazepines preoperatively and those who did not: patients using benzodiazepines were older, had a substantially higher comorbidity burden, and underwent the included procedures at different rates. While some differences are expected because the comorbidity is directly associated with benzodiazepine use or misuse (eg, neurologic, psychiatric, and substance use disorders), differences were also found for unrelated conditions. This finding implies that patients using benzodiazepines are sicker and that, despite adjusting for numerous potential observable confounders, the possibility of additional hidden confounders associated with benzodiazepine use is substantial. The limited nature of claims data also prevents adjustment for other factors, such as preoperative pain level, socioeconomic status, and the use of any benzodiazepine and opioid medications that were not filled using the patient’s insurance plan. Furthermore, as with all retrospective cohort studies, causality of identified potential risk factors cannot be determined.

## Conclusions

A growing body of literature suggests that postoperative outcomes can be improved by optimizing the management of comorbidities and opioid use during the preoperative period, including cardiovascular disease,^29^ diabetes,^30^ obesity,^31^ and smoking,^32^ as well as other factors.^33^ In the context of the increasingly deadly opioid epidemic, the results of this study appear to support the inclusion of discussions about the risks and benefits of continuing benzodiazepine therapy during the perioperative period as a component of preoperative optimization.

## Notes

Supplement. **eFigure 1.** Sample Population Flow Chart **eTable 1.** List of CPT Codes **eTable 2.** Benzodiazepine Conversion Factors to Calculate Diazepam Milligram Equivalents (DMEs) **eFigure 2.** Average Number of Prescriptions per Benzodiazepine Medication During Preoperative Year **eReferences.**

## References

[1] Centers for Disease Control and Prevention CDC WONDER: multiple cause of death data. Updated February 10, 2020. Accessed December 25, 2018. https://wonder.cdc.gov/mcd.html

[2] Scholl L, Seth P, Kariisa M, Wilson N, Baldwin G Drug and Opioid-Involved Overdose Deaths - United States, 2013-2017. MMWR Morb Mortal Wkly Rep. 2018;67(5152):1419-1427. doi:10.15585/mmwr.mm675152e1 30605448 PMC6334822

[3] Hah JM, Bateman BT, Ratliff J, Curtin C, Sun E Chronic opioid use after surgery: implications for perioperative management in the face of the opioid epidemic. Anesth Analg. 2017;125(5):1733-1740. doi:10.1213/ANE.0000000000002458 29049117 PMC6119469

[4] Wunsch H, Wijeysundera DN, Passarella MA, Neuman MD Opioids prescribed after low-risk surgical procedures in the United States, 2004-2012. JAMA. 2016;315(15):1654-1657. doi:10.1001/jama.2016.0130 26978756 PMC4837043

[5] Menendez ME, Ring D, Bateman BT Preoperative opioid misuse is associated with increased morbidity and mortality after elective orthopaedic surgery. Clin Orthop Relat Res. 2015;473(7):2402-2412. doi:10.1007/s11999-015-4173-5 25694266 PMC4457771

[6] Cron DC, Englesbe MJ, Bolton CJ, Preoperative opioid use is independently associated with increased costs and worse outcomes after major abdominal surgery. Ann Surg. 2017;265(4):695-701. doi:10.1097/SLA.0000000000001901 27429021

[7] Rozell JC, Courtney PM, Dattilo JR, Wu CH, Lee GC Preoperative opiate use independently predicts narcotic consumption and complications after total joint arthroplasty. J Arthroplasty. 2017;32(9):2658-2662. doi:10.1016/j.arth.2017.04.002 28478186

[8] Lembke A, Papac J, Humphreys K Our other prescription drug problem. N Engl J Med. 2018;378(8):693-695. doi:10.1056/NEJMp1715050 29466163

[9] Olfson M, King M, Schoenbaum M Benzodiazepine use in the United States. JAMA Psychiatry. 2015;72(2):136-142. doi:10.1001/jamapsychiatry.2014.1763 25517224

[10] Sun EC, Dixit A, Humphreys K, Darnall BD, Baker LC, Mackey S Association between concurrent use of prescription opioids and benzodiazepines and overdose: retrospective analysis. BMJ. 2017;356:j760. doi:10.1136/bmj.j760 28292769 PMC5421443

[11] Sigurdsson MI, Helgadottir S, Long TE, Association between preoperative opioid and benzodiazepine prescription patterns and mortality after noncardiac surgery. JAMA Surg. 2019;154(8):e191652. doi:10.1001/jamasurg.2019.1652 31215988 PMC6584895

[12] Gaulton TG, Wunsch H, Gaskins LJ, Preoperative sedative-hypnotic medication use and adverse postoperative outcomes. Ann Surg. 2019;1. doi:10.1097/SLA.0000000000003556 31415004 PMC7053280

[13] Sun EC, Darnall BD, Baker LC, Mackey S Incidence of and risk factors for chronic opioid use among opioid-naive patients in the postoperative period. JAMA Intern Med. 2016;176(9):1286-1293. doi:10.1001/jamainternmed.2016.3298 27400458 PMC6684468

[14] Hozack BA, Rivlin M, Lutsky KF, Preoperative exposure to benzodiazepines or sedative/hypnotics increases the risk of greater filled opioid prescriptions after surgery. Clin Orthop Relat Res. 2019;477(6):1482-1488. doi:10.1097/CORR.0000000000000696 31094846 PMC6554110

[15] Centers for Disease Control and Prevention Calculating total daily dose of opioids for safer dosage. Accessed December 25, 2018. https://www.cdc.gov/drugoverdose/pdf/calculating_total_daily_dose-a.pdf

[16] Dowell D, Haegerich TM, Chou R CDC guideline for prescribing opioids for chronic pain—United States, 2016. MMWR Recomm Rep. 2016;65(1):1-49. doi:10.15585/mmwr.rr6501e1 26987082

[17] Miller NS, Gold MS Management of withdrawal syndromes and relapse prevention in drug and alcohol dependence. Am Fam Physician. 1998;58(1):139-146. 9672434

[18] Ng K, Dahri K, Chow I, Legal M Evaluation of an alcohol withdrawal protocol and a preprinted order set at a tertiary care hospital. Can J Hosp Pharm. 2011;64(6):436-445. doi:10.4212/cjhp.v64i6.1085 22479099 PMC3242577

[19] Lieberman J. A. & Allan T. Handbook of Psychiatric Drugs. John Wiley & Sons Ltd; 2006.

[20] Ashton CH Benzodiazepines: how they work and how to withdraw. Updated August 2002 Accessed December 25, 2018. https://benzo.org.uk/manual/contents.htm

[21] Hewitt J. Calculating Equivalent Doses of Oral Benzodiazepines. National Heath Service Foundation Trust; 2014.

[22] Miller NS, Kipnis SS. Detoxification and Substance Abuse Treatment. Substance Abuse and Mental Health Services Administration; 2006. 22514851

[23] Quan H, Sundararajan V, Halfon P, Coding algorithms for defining comorbidities in *ICD-9-CM* and *ICD-10* administrative data. Med Care. 2005;43(11):1130-1139. doi:10.1097/01.mlr.0000182534.19832.83 16224307

[24] Elixhauser A, Steiner C, Harris DR, Coffey RM Comorbidity measures for use with administrative data. Med Care. 1998;36(1):8-27. doi:10.1097/00005650-199801000-00004 9431328

[25] Kisely S, Lin E, Gilbert C, Smith M, Campbell LA, Vasiliadis HM Use of administrative data for the surveillance of mood and anxiety disorders. Aust N Z J Psychiatry. 2009;43(12):1118-1125. doi:10.3109/00048670903279838 20001410

[26] Amemiya T. Regression analysis when the dependent variable is truncated normal. Econometrica. 1973;41(6):997-1016. doi:10.2307/1914031

[27] O’Donnell O, van Doorslaer E, Wagstaff A, Lindelow M. Analyzing Health Equity Using Household Survey Data. World Bank; 2008. doi:10.1596/978-0-8213-6933-3

[28] Sun EC, Dexter F, Miller TR, Baker LC “Opt out” and access to anesthesia care for elective and urgent surgeries among US Medicare beneficiaries. Anesthesiology. 2017;126(3):461-471. doi:10.1097/ALN.0000000000001504 28106610

[29] Fleisher LA, Fleischmann KE, Auerbach AD, 2014 ACC/AHA guideline on perioperative cardiovascular evaluation and management of patients undergoing noncardiac surgery: executive summary: a report of the American College of Cardiology/American Heart Association Task Force on Practice Guidelines. Circulation. 2014;130(24):2215-2245. doi:10.1161/CIR.0000000000000105 25085962

[30] Sebranek JJ, Lugli AK, Coursin DB Glycaemic control in the perioperative period. Br J Anaesth. 2013;111(suppl 1):i18-i34. doi:10.1093/bja/aet381 24335396

[31] Joshi GP, Ahmad S, Riad W, Eckert S, Chung F Selection of obese patients undergoing ambulatory surgery: a systematic review of the literature. Anesth Analg. 2013;117(5):1082-1091. doi:10.1213/ANE.0b013e3182a823f4 24108263

[32] Lindström D, Sadr Azodi O, Wladis A, Effects of a perioperative smoking cessation intervention on postoperative complications: a randomized trial. Ann Surg. 2008;248(5):739-745. doi:10.1097/SLA.0b013e3181889d0d 18948800

[33] Fong R, Sweitzer BJ Preoperative optimization of patients undergoing ambulatory surgery. Curr Anesthesiol Rep. 2014;4:303-315. doi:10.1007/s40140-014-0082-5

