# Supplementary material for: Association Between Preoperative Benzodiazepine Use and Postoperative Opioid Use and Health Care Costs
Source: JAMA Netw Open. 2020 Oct 27;3(10):e2018761. doi: 10.1001/jamanetworkopen.2020.18761 (PMC7592026; doi:10.1001/jamanetworkopen.2020.18761)
Supplement: Supplement. — eFigure 1. Sample Population Flow Chart eTable 1. List of CPT Codes eTable 2. Benzodiazepine Conversion Factors to Calculate Diazepam Milligram Equivalents (DMEs) eFigure 2. Average Number of Prescriptions per Benzodiazepine Medication During Preoperative Year eReferences. [file jamanetwopen-e2018761-s001.pdf]

Supplementary Online Content

Rishel CA, Zhang Y, Sun EC. Association between preoperative benzodiazepine use and postoperative opioid use and health care costs. *JAMA Netw Open*. 2020;3(10):e2018761. doi:10.1001/jamanetworkopen.2020.18761

eFigure 1. Sample Population Flow Chart

eTable 1. List of CPT Codes

eTable 2. Benzodiazepine Conversion Factors to Calculate Diazepam Milligram Equivalents (DMEs)

eFigure 2. Average Number of Prescriptions per Benzodiazepine Medication During Preoperative Year

eReferences

This supplementary material has been provided by the authors to give readers additional information about their work.

© 2020 Rishel CA et al. *JAMA Network Open.*

eFigure 1. Sample population flow chart

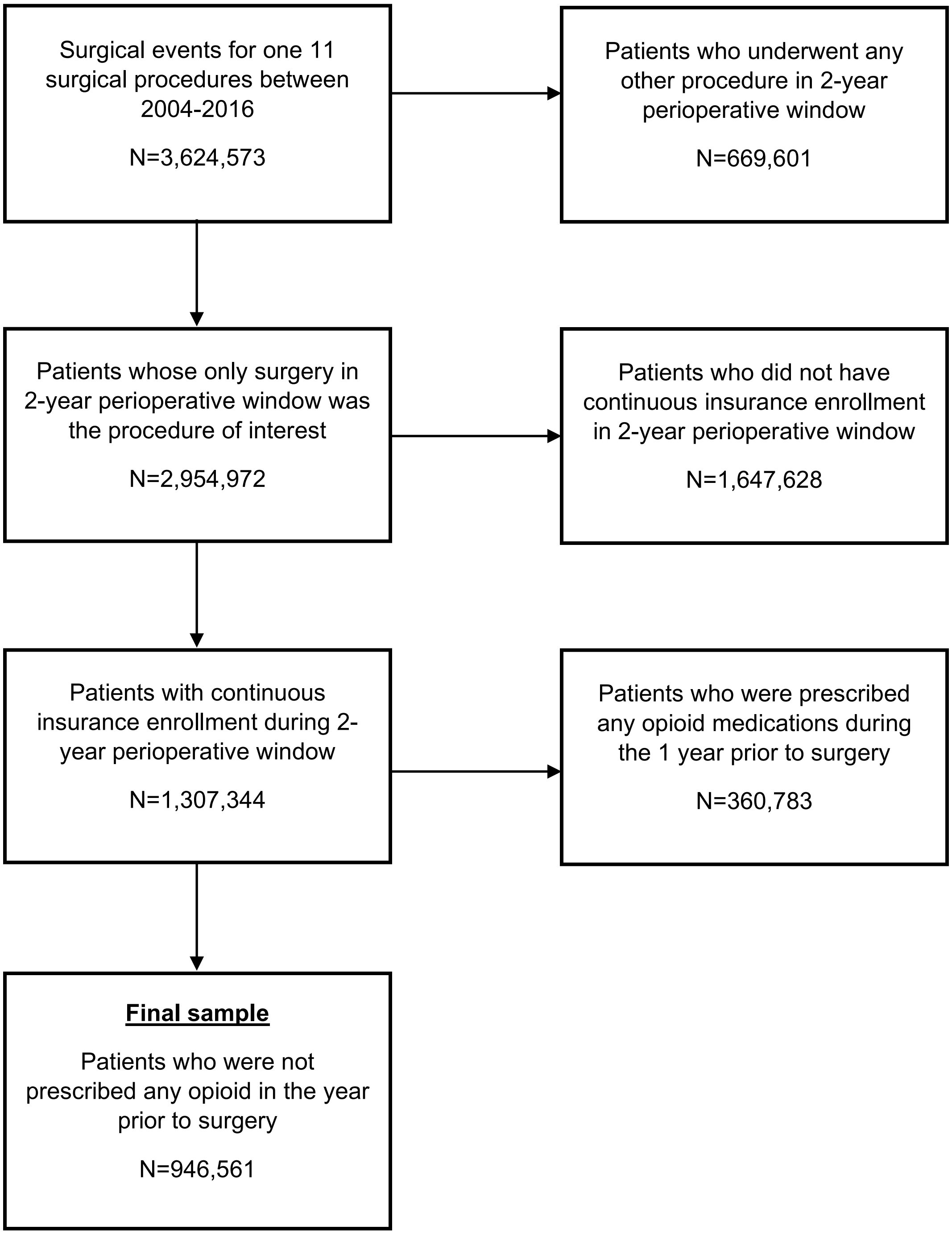

© 2020 Rishel CA et al. *JAMA Network Open.*

eTable 1. List of CPT codes[5]

Procedure name	CPT codes
Total Knee Arthroplasty	27447
Total Hip Arthroplasty	27130
Laparoscopic Cholecystectomy	47562, 47563, 47564
Open Cholecystectomy	47600, 47605, 47610
Laparoscopic Appendectomy	44970, 44979
Open Appendectomy	44950, 44960
Cesarean Section	59510, 59514, 59515
Functional Endoscopic Sinus Surgery	31237, 31240, 31254, 31255, 31256, 31267, 31276, 31287, 31288
Cataract Surgery	66982, 66983, 66984
Transurethral Resection of the Prostate	52601, 52612, 52614
Simple Mastectomy	19301, 19302, 19303, 19180

© 2020 Rishel CA et al. *JAMA Network Open.*

eTable 2. Benzodiazepine conversion factors to calculate diazepam milligram equivalents (DMEs)[1–4]

Generic name of benzodiazepine	Dose equal to 10 mg of diazepam**
Alprazolam	1 mg
Chlordiazepoxide	25 mg
Clonazepam	2 mg
Chlorazepate	7.5 mg
Diazepam	10 mg
Estazolam	1 mg
Flurazepam	15 mg
Lorazepam	2 mg
Oxazepam	10 mg
Quazepam	15 mg
Temazepam	15 mg
Triazolam	0.25 mg

© 2020 Rishel CA et al. *JAMA Network Open.*

eFigure 2. Average number of prescriptions per benzodiazepine medication during preoperative year

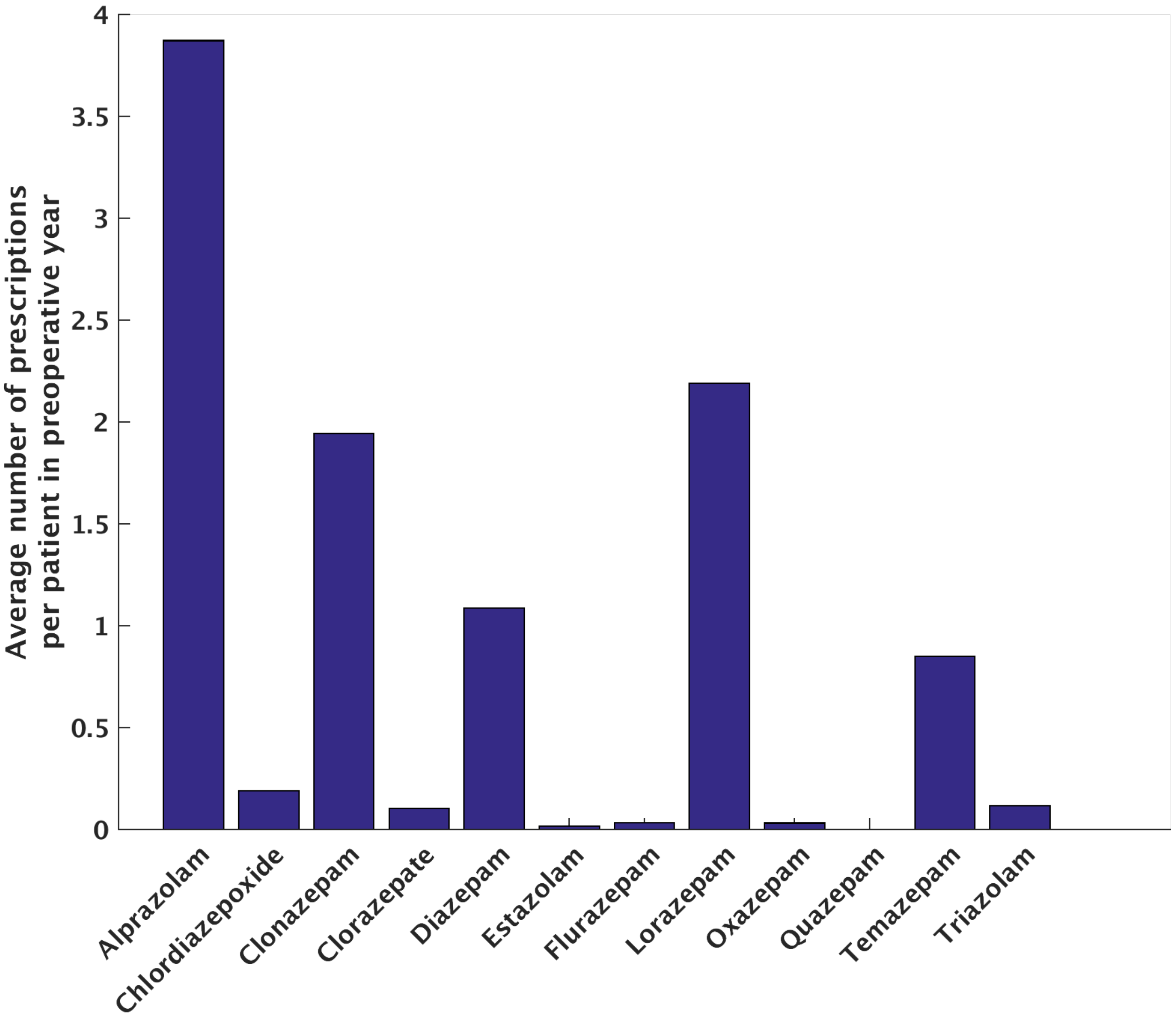

© 2020 Rishel CA et al. *JAMA Network Open.*

eReferences

1. Miller, N. S. & Kipnis, S. S. *Detoxification and Substance Abuse Treatment*. *Detoxification and Substance Abuse Treatment* (Substance Abuse and Mental Health Services Administration (US), 2006).
2. Miller, N. S. & Gold, M. S. Management of withdrawal syndromes and relapse prevention in drug and alcohol dependence. *Am. Fam. Physician* **58**, 139–46 (1998).
3. Lieberman, J. A. & Allan, T. *Handbook of Psychiatric Drugs*. (John Wiley & Sons Ltd., 2006).
4. Ashton, C. H. *Benzodiazepines: How They Work and How to Withdraw*. (2002).
5. Sun, E. C., Darnall, B. D., Baker, L. C. & Mackey, S. Incidence of and Risk Factors for Chronic Opioid Use Among Opioid-Naive Patients in the Postoperative Period. *JAMA Intern. Med.* **176**, 1286 (2016).
